# ZmNAC074, a maize stress-responsive NAC transcription factor, confers heat stress tolerance in transgenic *Arabidopsis*

**DOI:** 10.3389/fpls.2022.986628

**Published:** 2022-09-29

**Authors:** Yan Xi, Qiqi Ling, Yue Zhou, Xiang Liu, Yexiong Qian

**Affiliations:** Anhui Provincial Key Laboratory of Conservation and Exploitation of Important Biological Resources, College of Life Sciences, Anhui Normal University, Wuhu, China

**Keywords:** maize, heat stress, *ZmNAC074*, transcription factor, ROS homeostasis, stress tolerance

## Abstract

The harsh environment such as high temperature greatly limits the growth, development and production of crops worldwide. NAC (NAM, ATAF1/2, and CUC2) transcription factors (TFs) play key regulatory roles in abiotic stress responses of plants. However, the functional roles of NAC TFs in heat stress response of maize remain elusive. In our present study, we identified and isolated a stress-responsive NAC transcription factor gene in maize, designated as *ZmNAC074* and orthologous with rice *OsNTL3*. Further studies revealed that *ZmNAC074* may encode a membrane-bound transcription factor (MTF) of NAC family in maize, which is comprised of 517 amino acid residues with a transmembrane domain at the C-terminus. Moreover, *ZmNAC074* was highly expressed and induced by various abiotic stresses in maize seedlings, especially in leaf tissues under heat stress. Through generating *ZmNAC074* transgenic plants, phenotypic and physiological analyses further displayed that overexpression of *ZmNAC074* in transgenic *Arabidopsis* confers enhanced heat stress tolerance significantly through modulating the accumulation of a variety of stress metabolites, including reactive oxygen species (ROS), antioxidants, malondialdehyde (MDA), proline, soluble protein, chlorophyll and carotenoid. Further, quantitative real-time PCR analysis showed that the expression levels of most ROS scavenging and HSR- and UPR-associated genes in transgenic *Arabidopsis* were significantly up-regulated under heat stress treatments, suggesting that *ZmNAC074* may encode a positive regulator that activates the expression of ROS-scavenging genes and HSR- and UPR-associated genes to enhance plant thermotolerance under heat stress conditions. Overall, our present study suggests that *ZmNAC074* may play a crucial role in conferring heat stress tolerance in plants, providing a key candidate regulatory gene for heat stress tolerance regulation and genetic improvement in maize as well as in other crops.

## Introduction

According to previous studies, maize, rice, and wheat account for 89% of the global cereal production (Botticella et al., 2021). Maize, a crucial economic grain crop, contributes significantly to health and nutrition worldwide (Poole et al., 2020). The sixth assessment report from the IPCC points out that the surface temperature on the earth has increased by around 1.1°C compared with the average temperature from 1850 to 1900 and forecasts that the global average temperature will continue to rise (Tollefson, 2021). Global warming poses a major threat to human health and nutrition, seriously damaging crop growth, yield and food security (Guihur et al., 2022). Without adapting to the environment and human intervention, for every 1°C increase in global average temperature, maize yield will decrease by an average of 7.4% (Zhao et al., 2017). Previous studies have shown that the growth and development of maize has been affected by heat stress, such as inhibiting grain weight and starch deposition, reducing the pollen viability and the production of photosynthetic components, and the decline of nutrient absorption and yield traits (Lesk et al., 2016; Yang et al., 2018; Hussain et al., 2019; Wang et al., 2020; Li and Howell, 2021). Therefore, understanding the molecular regulatory mechanism underlying heat stress response in maize will enhance our ability to achieve agricultural productivity to the growing world population.

The survival of plants is seriously negatively affected by heat stress (Driedonks et al., 2016). Considering that plants are sessile organisms, the processes of plant growth and development largely depend on the effective activation of heat-tolerant regulatory networks to alleviate the damage resulted from heat stress response (Nievola et al., 2017). The mechanisms of plant heat stress response mainly involve heat shock response (HSR), unfolded protein response (UPR), ROS homeostasis, epigenetic controls and hormone-mediated regulation in plants (Liu et al., 2015; Ohama et al., 2017; Li et al., 2020a; Li et al., 2020b; Zhao et al., 2020). Studies have shown that numerous stress-associated defense genes and transcription factors (TFs) are involved in the regulation of heat stress response of plants, such as MAPK, CDPK, bZIP, and NAC TFs (Wu et al., 2015; Li et al., 2018; Li et al., 2020b; Liu et al., 2020; Zhao et al., 2021). Especially, TFs play a crucial role in regulating heat stress responses of plants. Numerous studies have shown that NAC TFs play different roles under heat stress in rice, *Arabidopsis* and other plants, such as OsNTL3, SNAC3, ANAC042, NAC019, and CaNAC035 (Shahnejat-Bushehri et al., 2012; Guan et al., 2014; Fang et al., 2015; Liu et al., 2020; Zhang et al., 2020). However, little is known regarding the roles of heat stress tolerance of NAC TFs in maize.

NAC (NAM, ATAF1/2, and CUC2) protein has been identified in Petunia for the first time (Souer et al., 1996). NAC family proteins share a highly conserved NAC-bound domain at the N-terminus and a variable and diverse transcriptional regulatory region at the C-terminus (Ooka et al., 2003; Olsen et al., 2005; Christianson et al., 2010). As one of the largest families of TFs in plants, NAC TFs play crucial roles in the regulation of plant growth, development and responses to biotic and abiotic stresses (Shao et al., 2015; Ohbayashi and Sugiyama, 2017; Mathew and Agarwal, 2018; Diao et al., 2020; Singh et al., 2021). For instance, rice *SNAC3* can enhance heat and drought resistance by regulating the dynamic balance of reactive oxygen species (ROS) (Fang et al., 2015). Overexpression of *ANAC087* gene in *Arabidopsis* can induce the increase of branching in inflorescence stems and involve in rosette development (Vargas-Hernández et al., 2022). Moreover, silencing *GhJUB1L1* gene can reduce drought tolerance and inhibit the development of secondary cell walls in cotton, suggesting that *GhJUB1L1* plays a positive regulatory role in the occurrence of drought stress tolerance and the development of secondary cell walls in cotton (Chen et al., 2021). Furthermore, ectopic overexpression of *ZmNAC126* in transgenic *Arabidopsis* and maize can enhance chlorophyll degradation and promote leaf senescence, suggesting that *ZmNAC126* plays a key role in regulating chlorophyll degradation during maize leaf senescence. Further, based on EMSA and CHIP-seq analyses, ZmNAC126 was revealed to directly bind to the promoters of major chlorophyll catabolic genes including *ZmNYE1*, *ZmNYC1*, *ZmPAO* and *ZmRCCR*, indicating that ZmNAC126 can positively regulate these chlorophyll catabolic genes in maize. Moreover, the expression of *ZmNAC126* can be induced by ethylene, and ZmEIN3, a major TF of ethylene signaling, can bind to its promoter to transactivate its expression during leaf senescence (Yang et al., 2020). In addition, the overexpression of *ZmNAC55* in *Arabidopsis* led to the sensitivity of transgenic *Arabidopsis* lines to ABA at germination stage, but improved the drought resistance compared with wild-type seedlings (Mao et al., 2016).

Recently, a considerable attention has been paid to the functional roles of NAC TFs in response to heat stress. For example, it has been revealed that OsNTL3 can interact with OsbZIP74 to activate the regulatory circuits of the nucleus, endoplasmic reticulum and plasma membrane under heat stress to enhance heat resistance of rice (Liu et al., 2020). *ONAC127* and *ONAC129* in rice have been displayed to respond to heat stress and regulate grain filling by modulating sugar transport and abiotic stress responses (Ren et al., 2021). Moreover, overexpression of *JUNGBRUNNEN1* (*JUB1*; *ANAC042*) could increase the longevity of *Arabidopsis* and enhance the tolerance to heat stress (Shahnejat-Bushehri et al., 2012). Overexpression of *TaNAC2L* can regulate the expression of heat stress-associated genes, thereby promoting heat resistance in transgenic *Arabidopsis* (Guo et al., 2015). Taken together, these studies have revealed that NAC TFs may play vital regulatory roles in dealing with heat stress responses of plants. At present, a total of 148 *NAC* genes (*ZmNAC1*-*ZmNAC148*) have been identified in maize (Peng et al., 2015). However, up to now, knowledge regarding the regulatory roles of NAC TFs in response to heat stress in maize remains scarce. In our previous studies, one transcript (*ZmNAC074*) has been identified through high- throughput sequencing analysis of maize B73 inbred line and exhibited significantly up-regulated expression level in leaves of maize seedling after heat stress treatment (Qian et al., 2019).

Previously, *ZmNAC074* (also known as *ZmNTL1* or *ZmNAC45*) was reported to encode a membrane-bound transcription factor of NAC family in maize and respond to oxidative stress (Shiriga et al., 2014; Lu et al., 2015; Wang et al., 2016). Generally, some TFs are immobilized on plasma membrane as MTFs and remain dormant under normal conditions, but these MTFs are likely to be released from the plasma membrane by proteolytic cleavage to become active TFs under stress conditions (Seo et al, 2008) , such as TaNTL1 (Sun et al., 2022), OsNTL3 (Liu et al., 2020), and ANAC062(NTL6) (Yang et al., 2014a). Previous studies have revealed that some NAC MTFs, such as OsNTL3, AtNAC062, AtNAC089 and AtNAC103, are associated with UPR, especially through interaction with the members of bZIP protein family (Sun et al., 2013; Yang et al., 2014a; Yang et al., 2014b; Liu et al., 2020). Therefore, we strive to reveal potential regulatory roles of ZmNAC074 as a MTF under heat stress treatments in this study.

Taken together, the goals of the present study were to validate the biological function of *ZmNAC074* in response to heat stress and to provide a molecular basis for using *ZmNAC074* to improve maize thermotolerance *via* transgenic and genome editing approaches. By characterizing the *ZmNAC074* overexpression transgenic *Arabidopsis* lines under heat stress treatments, we demonstrate that *ZmNAC074* may play a positive regulatory role in regulating heat resistance by modulating the accumulation of multiple stress metabolites and participating in the processes of HSR, UPR and ROS homeostasis. Overall, our study will provide an important theoretical basis for further improvement of heat resistance of maize or other crops through genetic engineering.

## Materials and methods

### Plant materials and growth conditions

In this study, the seeds of maize B73 Inbred Line were obtained from Anhui Agricultural University, China. The seeds germinated on the wet cotton cloth at 28°C for 3 days, and then the germinated seeds were transplanted to nutritious soil and grew at 28°C with long-day conditions of 15 h (550 μmol m^-2^s^-1^) and 9 h of dark and the relative humidity of 60% for the whole growth and developmental period. At the V4 developmental period (there is a visible ring in the leaf base of the fourth leaf), the maize seedlings were transferred to a dark incubator with 42°C treatment for 0, 4, and 8 h. Moreover, according to the previous methods (Hu et al., 2021), the seedlings were subjected to drought and salt treatments (200 mM NaCl) for 0, 4, and 8 h, respectively. The treated seedling roots, stems and leaves were collected after the three stress treatments, respectively. All these collected tissues were stored in liquid nitrogen to further extract RNA. In addition, *Arabidopsis* ecotype Columbia (Col) was used as original materials for genetic transformation to obtain transgenic plants and perform further functional analysis. All *Arabidopsis* plants were grown in the controlled chamber (22°C,16h light (120 μmol m^-2^s^-1^)/8 h darkness, and 60% relative humidity).

### Bioinformatic analysis of *ZmNAC074*

The full-length CDS sequence of *ZmNAC074* was extracted from the NCBI database (http://www.ncbi.nlm.nih.gov). The orthologous protein sequences in rice and *Arabidopsis* were retrieved from Phytozome (https://phytozome-next.jgi.doe.gov/) and NCBI (http://www.ncbi.nlm.nih.gov) (Zhang et al., 2022). Furthermore, the molecular weight and theoretical isoelectric point (PI) of ZmNAC074 protein were calculated in Expasy (http://web.expasy.org/compute_pi/) (Khan et al., 2018). The multiple amino acid sequences were calibrated and compared using ClustalW program. The phylogenetic tree was constructed using MEGA 7.0 software with the neighbor-joining method and 1000 bootstrap replicates in this study. The conserved motifs were predicted using online MEME website (http://alternate.meme-suite.org/tools/meme). In addition, the structure of these genes was analyzed by GSDS website (http://gsds.cbi.pku.edu.cn/) referring to our previous methods (Hu et al., 2021). The amino acid sequences of ZmNAC074 and OsNTL3 proteins were further compared using DNAMAN software. The transmembrane domain of ZmNAC074 was predicted using TMHHM 2.0 (https://services.healthtech.dtu.dk/service.php?TMHMM-2.0). The three dimensional (3D) structural model of ZmNAC074 protein was predicted using the I-TASSER server (https://seq2fun.dcmb.med.umich.edu//I-TASSER/) (Yang and Zhang, 2015; Zhang et al., 2017; Zheng et al., 2021). The predicted model was edited and annotated using the PyMOL software. Moreover, the functional associations of several NAC MTFs and stress response-associated proteins in *Arabidopsis* and rice were predicted to construct their protein protein interaction networks by the STRING website (https://cn.string-db.org/), respectively.

### Quantitative real-time PCR analysis

The total RNA was isolated with Trizol reagent (TIANGEN, China) (Gai et al., 2020). The first strand of cDNA was synthesized from 2μg of total RNA using the FastKing RT Kit (TIANGEN, China). Quantitative real time-PCR (qRT-PCR) was performed using Light-CykerR96 real-time PCR system (BioRad, USA). The reaction conditions are as follows: preincubation at 95°C for 600 s, and then performing 40 cycles of 95°C for 10s, 55°C for 30s and 72°C for 30s. In maize and *Arabidopsis*, the relative gene expression data were calculated using the 2^−△△Ct^ method with the expression of *ZmActin* and *AtActin* as internal controls, respectively. All qRT-PCR analyses were performed by three biological replicates. The primer sequences used were listed in **Supplementary Table S1**.

### Vector construction and *Arabidopsis* transformation

The full-length CDS sequence of *ZmNAC074* was amplified by PCR and inserted into the PHB vector driven by the cauliflower mosaic virus (CaMV) 35S promoter to generate a recombinant vector, which was confirmed *via* double digestion validation with Hind III and Xba I enzymes and sequencing. The constructed vector was further transformed into *Arabidopsis* ecotype Col-0 using the floral dipping method mediated by *Agrobacterium tumefaciens* strain GV3101. The seeds of T_0_ and T_1_ generations were screened with 1/2 MS media containing 30 mg/L hygromycin and identified by RT-PCR. Three T_3_ homozygotes of *ZmNAC074* transgenic overexpression lines (line 1, line 2, and line 9) were ultimately selected for further functional analysis (Gai et al., 2020).

### Heat stress treatment

The three-week-old transgenic *Arabidopsis* and WT *Arabidopsis* seedlings were treated at 42°C for 24h and 48h, respectively (Huang et al., 2019). Then, these two kinds of *Arabidopsis* plants with different treatment time were restored to normal environmental conditions growing for one week. The determination of physiological characteristics and related gene expression was performed just at the end of heat stress treatments.

### Physiological and enzymatic analyses of transgenic *Arabidopsis* plants

Hydrogen peroxide (H_2_O_2_) in leaves was determined with 3, 3’-diaminobenzidine (DAB) solution. According to the previously described methods (Daudi and O’brien, 2012), the leaves were immersed in 1.0 mg/mL DAB-HCl solution (add the mixed solution of 0.05% (v/v) Tween 20 and 8 times the volume of 200 mM Na_2_HPO_4_ to adjust the PH of DAB-HCl solution to 7.0) for 8 h at 25 °C and dark. Then place the stained leaves in a beaker containing 95% (v/v) alcohol and carefully place them in a boiling water bath (95°C) for 15 ± 5 minutes until the brown spots appear on the leaves and there are no green areas (Ali et al., 2020). Further, the histochemical staining of superoxide radical (O_2_^-^) was mentioned as previously described (Xia et al., 2009; Liu et al., 2012), but with slight modifications. The leaves were incubated in 0.1% nitro blue tetrazolium (NBT) with 50 mM potassium phosphate buffer (pH 7.8) at 25°C for 12 h. Then, the same decolorization method mentioned above was used for bleaching and photography.

The sample pretreatment methods for some physiological indexs (MDA, soluble protein and proline) in this experiment are as follows: weigh 0.1g of leaves, add 900 mL of physiological saline, mechanically homogenize in an ice-water bath, centrifuge at 10,000 rpm for 10 min and take the sample supernatant for subsequent experiments. The activities of superoxide dismutase (SOD) and catalase (CAT) were determined using xanthine-xanthine oxidase method and ammonium molybdate spectrophotometric method, respectively (Hadwan and Abed, 2016). SOD and CAT activities were calculated by the absorbance value obtained at the wavelength of 550 nm and 405 nm, respectively. Moreover, the total antioxidant capacity (T-AOC) was determined by colorimetry at 520nm as directed by the manufacturer. In addition, the H_2_O_2_ content was determined with molybdenic acid colorimetry. H_2_O_2_ bound with molybdenic acid to form a complex (Zhou et al., 2021), which was measured by using spectrophotometer at the wavelength of 405nm. Then, the content of H_2_O_2_ was calculated from the measured absorbance. Malondialdehyde (MDA) content was determined using the thiobarbituric acid (TBA) assay (Aguilar Diaz De Leon and Borges, 2020). For this, 0.1mL of the sample supernatant was mixed with 4.1mL of the mixed solution containing TBA configured according to the manufacturer’s instructions and heated at 95°C for 40min. The reaction mixture was taken to determine the absorbance at 532nm and calculate the content of MDA. The content of soluble protein was determined by Coomassie Brilliant Blue method (Goldring, 2019) through adding 50μL the sample supernatant to 3mL Coomassie Brilliant Blue dye reagent, which was mixed and incubated at 25°C for 10 minutes. The graph of absorbance readings and soluble protein content at 595nm was plotted. The content of soluble protein was calculated by using the absorbance value of the reaction mixture and the graph. Furthermore, the proline content in leaves was determined by acid ninhydrin method (Shabnam et al., 2016). 0.5mL of sample supernatant mixed with 2mL of 1.25% (v/v) ninhydrin in 2mL glacial acetic acid at 100°C for 30 min. After cooling, the reaction mixture was taken to determine the absorbance at 520 nm. Moreover, to identify the content of photosynthetic pigments, 1g leaf tissue (ground with liquid nitrogen) was treated with 80% (v/v) acetone. The absorbance was measured at 470nm, 645nm, and 633nm, respectively (Zengin, 2013). Using the absorbance value, the contents of chlorophyll and carotenoid were calculated, respectively. All analyses were performed for three biological replicates. All the above kits or reagents were purchased from Nanjing Jiancheng Bioengineering Institute. Spectrophotometer (Shanghai Youke instrument Co., Ltd., UV752N) was used for all measurements.

### Statistical analysis

Statistical analysis was carried out using the SPSS 20 software. The data were analyzed by one-way ANOVA and the means were compared by Duncan’s multiple range test. Compared with the control group, P < 0.05 is considered to be statistically significant and P < 0.01 extremely significant.

## Results

### Sequence characterization of ZmNAC074 as a typical NAC membrane-bound TF

**Figure 1:**
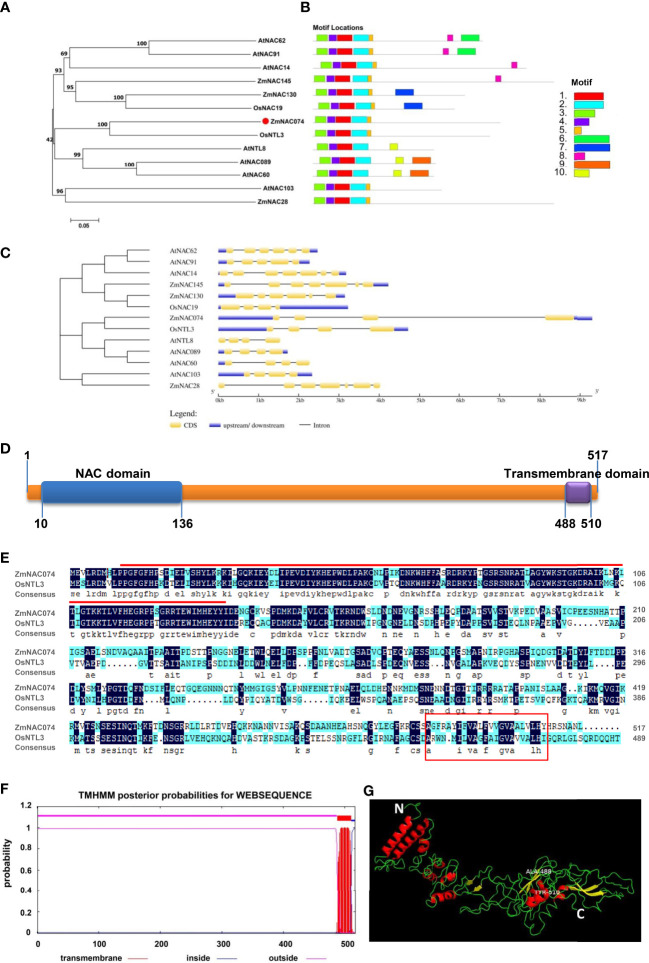
Bioinformatics analysis of the ZmNAC074 protein. **(A)** The phylogenetic tree of ZmNAC074 and other different NAC proteins. Bootstrap support values are indicated on each node. **(B)** The conserved motifs of these NAC proteins. **(C)** The gene structures of these NAC genes. **(D)** The domain distribution in ZmNAC074 protein. **(E)** Alignment of protein sequences of ZmNAC074 and OsNTL3. Red line represents NAC domain and red box represents transmembrane region. **(F)** The prediction of secondary transmembrane domain. **(G)** The 3D structural model of ZmNAC074 protein. N and C represent the N-terminus and C-terminus of ZmNAC074 protein, respectively.

The open reading frame (ORF) of *ZmNAC074* is consisted of 1556 bp, which encodes 517 amino acids (AA) with a molecular weight of 57.5 kDa and a theoretical isoelectric point (PI) of 5.49. It is suggested that ZmNAC074 protein is a kind of acidic protein and under the condition of isoelectric point (PI=5.49), this protein shows its minimum solubility, viscosity, osmotic pressure and swelling property. The phylogenetic tree of NAC proteins in maize, rice, and *Arabidopsis* revealed that ZmNAC074 protein shares a close evolutionary relationship with rice OsNTL3 protein **(** **Figure 1A** **)**. In addition, AtNAC089, AtNAC60 and AtNTL8 also belong to the same branch with ZmNAC074, indicating that the evolutionary relationships among them are relatively conserved. Information of corresponding genes encoding these NAC proteins in plants was listed in **Supplementary Table S2**. According to sequence characteristics of these NAC proteins, 10 conserved motifs (motif 1-10) were predicted using the MEME program **(** **Figure 1B** **)**. All NAC proteins contain motifs 1-4. Except for AtNAC089, AtNAC60, and AtNTL8, all other proteins contain motif 5. It is worth noting that ZmNAC074 and OsNTL3 contain the same motif distribution (motif 1-5). Annotations of the motifs are listed in **Supplementary Table S3**. In addition, the corresponding genes encoding these NAC proteins (including ZmNAC074, OsNTL3, AtNAC089, AtNAC60 and AtNTL8) in the same branch share a common feature in gene structure, namely only containing three introns **(** **Figure 1C** **)**. Moreover, the N-terminus and C-terminus of ZmNAC074 protein contain a NAC domain (10-136 AA) and a transmembrane domain (488-510 AA), respectively **(** **Figure 1D** **)**. Moreover, the amino acid sequence alignment between ZmNAC074 and OsNTL3 showed that their NAC domains share high similarity, indicating that ZmNAC074 may have a similar function to OsNTL3 **(** **Figure 1E** **)**. In addition, according to the TMHMM prediction, ZmNAC074 protein only contains one transmembrane domain, which is distributed at the C-terminus of ZmNAC074 protein **(** **Figure 1F** **)**. The positions of the N- terminus (represented by the white letter N) and the C- terminus (represented by the white letter C) of ZmNAC074 protein are marked in the picture of the 3D model **(** **Figure 1G** **)**. In addition, the middle region of the two labeled amino acids (ALA-488 and TYR-510) represents the transmembrane domain of this protein. Moreover, the α-helix and β-strand are depicted as red and yellow ribbon diagrams, respectively, and loops as green lines.

### *ZmNAC074* is responsive to various abiotic stresses in maize

**Figure 2:**
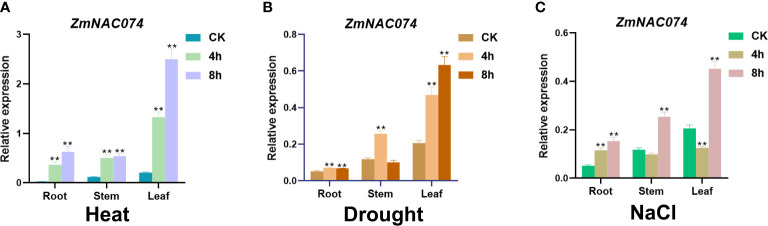
Expression pattern analyses of *ZmNAC074* in maize seedlings under various abiotic stress treatments. Expression levels of *ZmNAC074* in maize root, stem and leaf tissues after treatment with 42°CC **(A)**, drought **(B)**, and 200 Mm NaCl **(C)**, respectively. *ZmActin* was used as an internal control. The error bar is calculated through three biological replications. Asterisks above the error bar indicate significant differences (**, P < 0.01) using one-way ANOVA and a Fisher’s least significant difference (LSD).

To examine whether *ZmNAC074* responds to heat stress and other abiotic stresses, the expression pattern of *ZmNAC074* was evaluated under heat (42°C), salt (200mM NaCl), and drought stress treatments for 0, 4, and 8 h in maize B73 inbred line, respectively. The results showed that *ZmNAC074* was expressed in roots, stems, and leaves under normal conditions, but its expression level was the highest in leaves in comparison to other tissues **(** **Figure 2** **)**. The expression level of *ZmNAC074* was significantly upregulated following heat, drought and salt stress treatments, especially under heat stress treatment, respectively **(** **Figures 2A–C** **)**. After heat stress, the expression level of *ZmNAC074* in three different tissues increased gradually with the prolongation of stress time **(** **Figure 2A** **)**. After drought stress, the expression levels of *ZmNAC074* in stem and root tissues gradually increased, peaked at 4h, and then decreased **(** **Figure 2B** **)**. After salt stress, the expression levels of *ZmNAC074* in stem and root tissues decreased at 4h and then increased at 8h **(** **Figure 2A** **)**. Overall, these results demonstrate that *ZmNAC074* can respond to heat, drought, and salt stress, but the response to heat stress is particularly strong. Therefore, the regulatory role of *ZmNAC074* in heat stress tolerance was focused on in the subsequent study.

### Generation of *ZmNAC074*-overexpressed transgenic *Arabidopsis*

**Figure 3:**
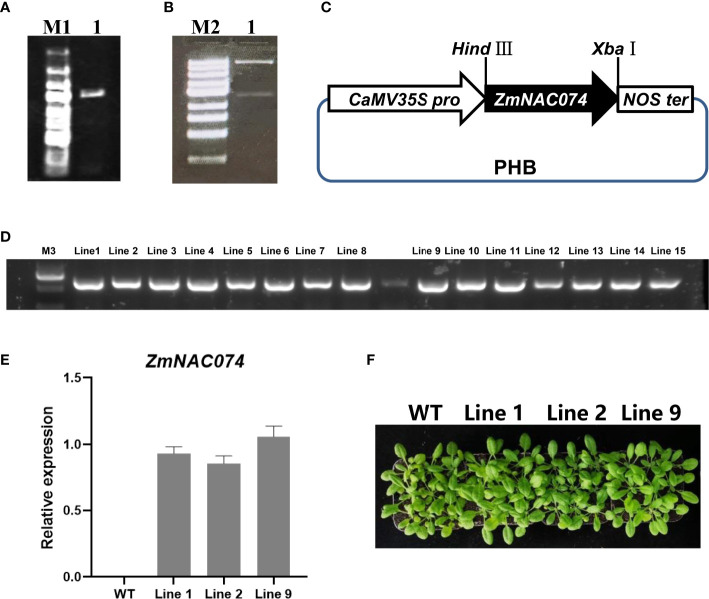
Generation and characterization of transgenic *Arabidopsis* plants overexpressing *ZmNAC074*. **(A)** Amplification of full-length CDS of *ZmNAC074*. Marker 1: 5k, 3k, 2k, 1500, 1000, 750, 500, 250, 100 (bp). **(B)** Enzyme validation of recombinant vector with *ZmNAC074* successfully inserted into the PHB vector. Marker 2: 10k, 5k, 3k, 2k, 1500, 1000, 750, 500, 250bp. **(C)** Schematic diagram of recombinant vector construction. **(D)** Detection of hygromycin resistance gene by PCR. Marker 3: 2k, 1000, 750, 500, 250, 100 (bp). **(E)** The qRT-PCR analysis of transgenic *Arabidopsis* plants overexpressing *ZmNAC074*. **(F)** The phenotype analysis of three transgenic *Arabidopsis* lines and WT *Arabidopsis* plants. The error bar is calculated through three biological replications. Asterisks above the error bar indicate significant differences using one-way ANOVA and a Fisher’s least significant difference (LSD).

To investigate the potential functions of *ZmNAC074* under heat stress, *ZmNAC074* was successfully introduced into *Arabidopsis* driven by *CaMV 35S* promoter **(** **Figures 3A–B** **)** (Zuo et al., 2022). The schematic diagram of constructed vector containing *ZmNAC074* under the control of *CaMV 35S* promoter was displayed **(** **Figure 3C** **)**. The recombinant vector was further transformed into *Arabidopsis* and 16 *ZmNAC074*-overexpressed transgenic *Arabidopsis* lines were obtained in this study. The amplification map showed that the exogenous *ZmNAC074* has been integrated into the genome of 15 transgenic *Arabidopsis* lines **(** **Figure 3D** **)**. Quantitative RT-PCR analysis showed that there was no significant difference in the expression of *ZmNAC074* among line 1, line 2, and line 9 when they were three weeks old **(** **Figure 3E** **)**. Moreover, when growing under normal conditions, there was no visible phenotypic difference between the three lines and WT *Arabidopsis* **(** **Figure 3F** **)**. Therefore, line 1, line 2 and line 9 can be selected as three biological repeats for further exploration.

### Overexpression of *ZmNAC074* affects the accumulation of stress-related metabolites during heat stress response

**Figure 4:**
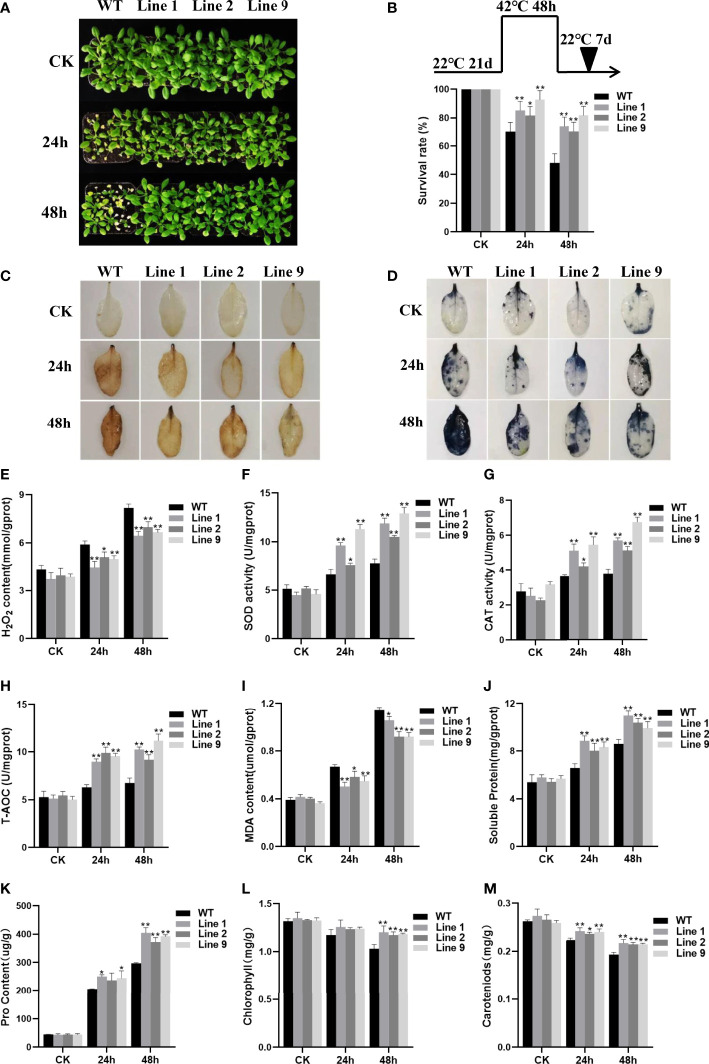
Analysis of heat resistance of transgenic plants overexpressing *ZmNAC074*. **(A)** Transgenic and WT *Arabidopsis* following heat stress treatment at 42°C for 24 h and 48h, respectively, and recovery for one week under 22°C normal conditions. Plants continually grown at 22°C were used as controls. **(B)** Survival rates of transgenic *Arabidopsis* lines and WT *Arabidopsis* plants after heat stress treatments. **(C, D)** H_2_O_2_ and O^2-^ accumulation was detected by using histochemical staining with DAB and NBT, respectively. **(E)** H_2_O_2_ contents in leaves of transgenic *Arabidopsis* lines and WT *Arabidopsis* plants before and after heat stress treatments. **(F–H)** Changes in activities of antioxidant enzymes SOD and CAT and the total antioxidant capacity in leaves of transgenic *Arabidopsis* lines and WT *Arabidopsis* plants before and after heat stress treatments. **(I–K)** Analyses of MDA, soluble protein and proline contents in leaves of transgenic *Arabidopsis* lines and WT *Arabidopsis* plants before and after heat stress treatments. **(L, M)** The leaf chlorophyll and carotenoid contents of transgenic *Arabidopsis* lines and WT *Arabidopsis* plants before and after heat stress treatments. *AtActin* was used as an internal control. The error bar is calculated through three biological replications. Asterisks above the error bar indicate significant differences (P < 0.05, P < 0.01) using one-way ANOVA and a Fisher’s least significant difference (LSD).

To further verify whether *ZmNAC074* is associated with heat resistance, three-week-old WT *Arabidopsis* and selected transgenic *Arabidopsis* lines were subjected to heat stress treatments for 24h and 48h, respectively. No visible difference was observed between WT *Arabidopsis* and transgenic *Arabidopsis* lines under normal conditions **(** **Figure 4A** **)**. However, under heat stress conditions, after all treated plants were transferred to normal growth environmental conditions and recovered for one week, the WT *Arabidopsis* leaves showed serious wilting symptoms, whereas numerous transgenic *Arabidopsis* leaves remained green **(** **Figure 4A** **)**. Notably, the phenotypic difference between WT *Arabidopsis* and transgenic *Arabidopsis* plants under heat stress treatment for 48h was more significant than that for 24h **(** **Figure 4A** **)**. According to statistics, after heat treatment for 24h, the survival ratio of transgenic *Arabidopsis* lines were 1.16-1.32 times higher than that of WT *Arabidopsis* plants, respectively. In contrast, after heat treatment for 48h, the survival ratio of all transgenic *Arabidopsis* lines was more than 70%, whereas that of WT *Arabidopsis* was less than 50% **(** **Figure 4B** **)**. The results indicated that transgenic *Arabidopsis* exhibited more obvious thermotolerance than WT *Arabidopsis.*

Furthermore, to investigate whether the heat tolerance phenotype of transgenic *Arabidopsis* lines was resulted from alteration of ROS homeostasis, the accumulation of ROS in transgenic *Arabidopsis* lines and WT *Arabidopsis* plants under normal and heat stress conditions was compared, respectively. Qualitative staining by DAB and NBT and quantitative measurement showed that the staining of H_2_O_2_ and superoxide anion (O_2_^-^) and the accumulation of H_2_O_2_ in leaf tissues of transgenic *Arabidopsis* lines were indistinguishable from those in WT *Arabidopsis* plants grown under normal conditions **(** **Figures 4C–E** **)**. However, after heat stress treatment, the staining of H_2_O_2_ and superoxide anion (O_2_^-^) was more evident and the accumulation of H_2_O_2_ was revealed to significantly increase in leaf tissues of transgenic *Arabidopsis* lines and WT *Arabidopsis* plants than that of corresponding control plants growing under normal conditions **(** **Figures 4C–E** **)**. Although the accumulation of ROS in all plants increased with the prolongation of heat stress treatment time, the staining of H_2_O_2_ and superoxide anion (O_2_^-^) in leaf tissues of transgenic *Arabidopsis* lines was less evident than that of WT *Arabidopsis* plants when treated with heat stress treatment for 24h and 48h, respectively. Accordingly, the transgenic *Arabidopsis* lines showed lower H_2_O_2_ accumulation levels than WT *Arabidopsis* plants. Thus, these results suggest that the cellular oxidative damage of transgenic *Arabidopsis* lines is lesser than that of WT *Arabidopsis* plants. Further, to detect the ability of plants to scavenge ROS, the activities of related SOD and CAT antioxidant enzymes and total antioxidant capacity (T-AOC) were evaluated in this study. Under normal conditions, there were no obvious differences in the SOD and CAT activities and T-AOC between the WT *Arabidopsis* and transgenic *Arabidopsis* lines. However, with the extension of heat stress time, the SOD and CAT activities and T-AOC of all plants were exhibited to increase, but the SOD and CAT activities and T-AOC of transgenic *Arabidopsis* lines were much higher than those of WT *Arabidopsis* plants **(** **Figures 4F–H** **)**. Thus, these results suggest that heat stress resistance of *ZmNAC074*-overexpressed transgenic *Arabidopsis* lines may be resulted from their enhanced ROS-scavenging capability.

Moreover, MDA (Malondialdehyde) can severely damage plant cell membranes, and thereby the degree of MDA accumulation is usually regarded as an important indicator of membrane-lipid peroxidation (Davey et al., 2005). In this study, no differences were observed in accumulation of MDA contents between all plants under normal conditions. However, after heat stress treatment, MDA contents of transgenic *Arabidopsis* lines were significantly lower than in WT *Arabidopsis* plants (**Figure 4I**). Therefore, these results suggest that overexpression of *ZmNAC074* in transgenic *Arabidopsis* may weaken the membrane lipid peroxidation and enhance the stability of cell membrane under heat stress, thereby contributing to slow down the oxidative damage caused by heat stress. In addition, compared with WT *Arabidopsis* plants, heat stress increased the accumulation of osmotic adjustment substances, including soluble proteins and proline in transgenic *Arabidopsis* lines (**Figures 4J, K**). Moreover, without heat stress treatment, the leaf chlorophyll and carotenoid contents of transgenic *Arabidopsis* lines were comparable to those of WT *Arabidopsis* plants **(** **Figures 4L, M** **)**. Interestingly, after 24h of heat stress treatment, there was no significant difference in chlorophyll content between transgenic *Arabidopsis* lines and WT *Arabidopsis* plants, but the carotenoid content of transgenic *Arabidopsis* lines was 1.05-1.08 times higher than that of WT *Arabidopsis* plants. Moreover, after 48h of heat stress treatment, the leaf chlorophyll and carotenoid contents of transgenic *Arabidopsis* lines were 1.14-1.17 and 1.11-1.13 times higher than those of WT *Arabidopsis* plants, respectively. Therefore, these results demonstrate that transgenic *Arabidopsis* lines can enhance heat stress tolerance by retaining more chlorophyll and carotenoids to protect photosystem II under heat stress.

### Overexpression of *ZmNAC074* alters expression patterns of heat stress responsive genes

**Figure 5:**
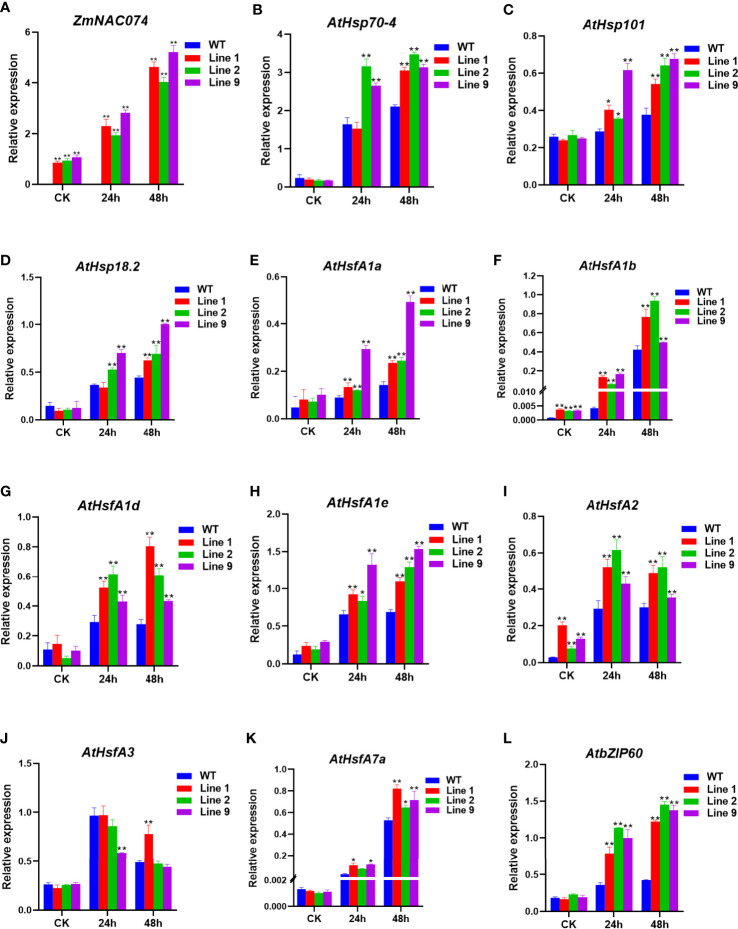
Expression levels of *ZmNAC074* and stress-responsive genes by qRT-PCR in transgenic *Arabidopsis* lines and WT *Arabidopsis* plants before and after heat stress treatments. Three-week-old transgenic *Arabidopsis* lines and WT *Arabidopsis* plants were exposed to heat stress (42°C) for 24 h and 48 h, and leaves were sampled for RNA extraction, cDNA synthesis and qPCR analysis. Gene-specific primers were used for detection of relative expression levels of stress-responsive genes, the raw date were normalized using *AtActin* as an internal control. For each experiment, three technical replicates were conducted. **(A–L)** The expression levels of *ZmNAC074*, *AtHsp70-4*, *AtHsp101*, *AtHsp18.2*, *AtHsfA1a-e*, *AtHsfA2*, *AtHsfA3*, *AtHsfA7a* and *AtbZIP60* were determined by qRT-PCR. *AtActin* was used as an internal control. The error bar is calculated through three biological replications. Asterisks above the error bar indicate significant differences (*P < 0.05, **P < 0.01) using one-way ANOVA and a Fisher’s least significant difference (LSD).

To elucidate the possible regulatory roles of *ZmNAC074* in heat stress tolerance, the expression levels of *ZmNAC074* and heat stress responsive genes have been characterized by qRT-PCR **(** **Figure 5A** **)**. There was no significant difference in the expression level of *ZmNAC074* among three transgenic *Arabidopsis* lines under normal conditions and after heat stress treatment for 24h. However, after 48h of heat stress treatment, the expression level of *ZmNAC074* in line 9 was higher than those in line 1 and line 2. It was worth noting that with the prolongation of heat stress treatment time, the expression level of *ZmNAC074* gradually increased. In addition, the expression levels of 11 heat stress responsive genes (*AtHsp70-4*, *AtHsp101*, *AtHsp18.2*, *AtHsfA1a-e*, *AtHsfA2*, *AtHsfA3*, *AtHsfA7a*, and *AtbZIP60*) were detected **(** **Figures 5B–L** **)**. Under normal conditions, except that the expression levels of *AtHsfA1b* and *AtHsfA2* genes in transgenic *Arabidopsis* lines were significantly higher than those in WT *Arabidopsis* plants, there was no significant difference in the expression levels of other genes between transgenic *Arabidopsis* lines and WT *Arabidopsis* plants under normal conditions. In contrast, under heat stress treatment, except that the expression level of *AtHsfA3* gene in transgenic *Arabidopsis* lines was lower than that in WT *Arabidopsis* plants, the expression levels of other genes in transgenic *Arabidopsis* lines were higher than those in WT *Arabidopsis* plants. Thus, these results suggest that *ZmNAC074* may enhance heat stress tolerance by regulating heat stress responsive genes.

### Overexpression of *ZmNAC074* affects the expression levels of genes associated with ROS scavenging

**Figure 6:**
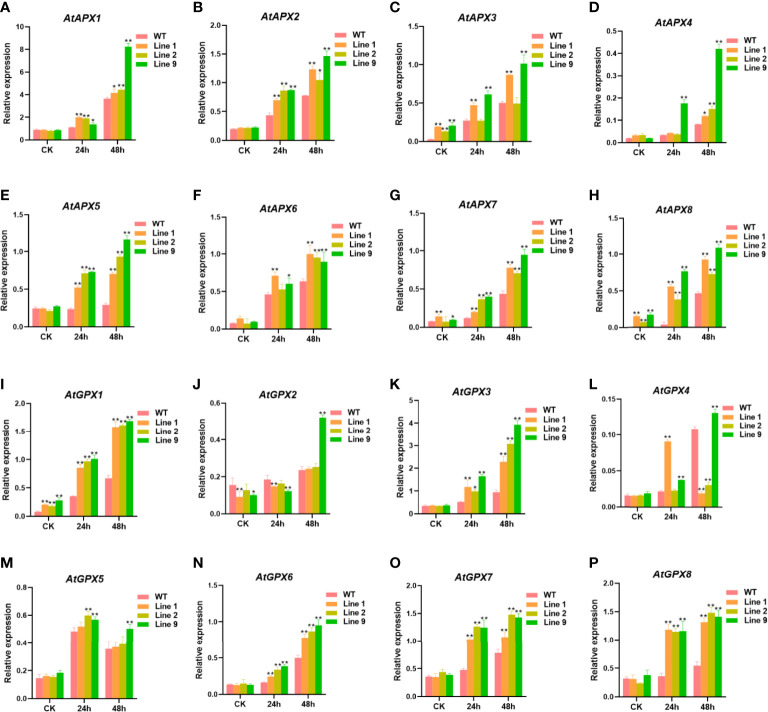
Expression levels of antioxidant-associated genes in transgenic *Arabidopsis* lines and WT *Arabidopsis* plants before and after heat stress treatments. Three-week-old transgenic *Arabidopsis* lines and WT *Arabidopsis* plants were exposed to heat stress (42°C) for 24 h and 48 h, and leaves were sampled for RNA extraction, cDNA synthesis and qPCR analysis. Gene-specific primers were used for detection of relative expression levels of stress-responsive genes, the raw date were normalized using *AtActin* as an internal control. For each experiment, three technical replicates were conducted. **(A–P)** The expression levels of *AtAPX1-8* and *AtGPX1-8* were determined by qRT-PCR. *AtActin* was used as an internal control. The error bar is calculated through three biological replications. Asterisks above the error bar indicate significant differences (*P < 0.05, **WFI 2P < 0.01) using one-way ANOVA and a Fisher’s least significant difference (LSD).

To further detect the ability of the *ZmNAC074*-overexpressed transgenic *Arabidopsis* to scavenge ROS, the expression levels of some antioxidant-related genes (*AtAPX1-8* and *AtGPX1-8*) between transgenic *Arabidopsis* lines and WT *Arabidopsis* plants under normal conditions and heat stress treatments were determined, respectively **(** **Figures 6A–P** **)**. Under normal conditions, there were no significant differences in the expression levels of most antioxidant-related genes between transgenic *Arabidopsis* lines and WT *Arabidopsis* plants, except for *AtAPX3*, *AtAPX7*, *AtAPX8*, *AtGPX1*, and *AtGPX2*. However, under heat stress treatments, the expression levels of most antioxidant-related genes in transgenic *Arabidopsis* lines were significantly higher than those in WT *Arabidopsis* plants. Noticeably, the expression levels of *AtGPX2* in three transgenic *Arabidopsis* lines was lower than that in WT *Arabidopsis* plants after heat stress treatment for 24h, and only transgenic line 9 exhibited higher *AtGPX2* expression level than WT *Arabidopsis* plants after heat stress treatment for 48h. Interestingly, the expression levels of most antioxidant-related genes were the highest in transgenic line 9 and the lowest in transgenic line 2. Taken together, these results suggest that overexpression of *ZmNAC074* in transgenic *Arabidopsis* can upregulate the expression levels of antioxidant-related genes under heat stress treatments.

### Protein protein interaction networks between several NAC MTFs and stress response- associated proteins in *Arabidopsis* and rice

**Figure 7:**
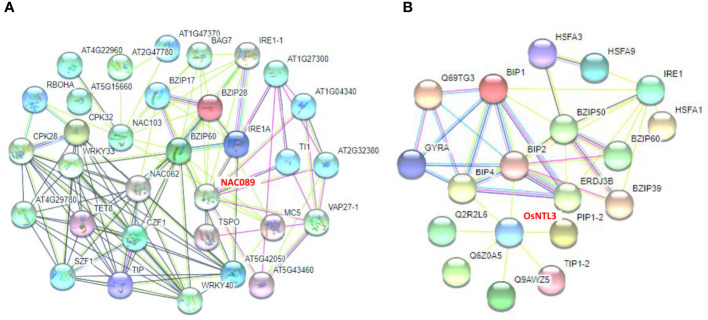
Protein Protein Interaction networks of NAC MTFs and stress response-associated proteins. **(A)** Functional association networks of NAC MTFs and stress response-associated proteins in *Arabidopsis.* AtNAC089 (shown in red font as NAC089) was highlighted to interact with the endoplasmic reticulum (ER) associated protein IRE1A. **(B)** Functional association networks of OsNTL3 (shown in red font) and heat or ER stress response-associated proteins in rice. OsNTL3 was highlighted to interact with BIP2 (a heat shock 70 kDa protein that functions as chaperone during ER stress response). Thick lines indicate high interaction.

To reveal the potential interaction effects of ZmNAC074 in response to heat stress, the possible interactions between some NAC MTFs and stress response-associated proteins in *Arabidopsis* and rice were integrated, especially OsNTL3 and AtNAC089, which are closely related to ZmNAC074 protein. The analysis of protein protein interaction network in *Arabidopsis* showed that bZIP family members, especially bZIP60, may be the central regulators of functional association network, which can interact with three NAC MTFs (including AtNAC089, AtNAC62, and AtNAC103) **(** **Figure 7A** **)**. Further, the analysis of protein protein interaction network in rice displayed that BIP2 and BIP4 can interact with OsNTL3 **(** **Figure 7B** **)**. Moreover, BIP2 and BIP4 are also closely related to bZIP family members, indicating the interaction between HSR-associated proteins and bZIP proteins. These networks are extremely complex, and there may be a variety of hierarchical regulation of NAC proteins in plants under heat or ER stress, such as the possibility of the interaction between OsNTL3 and BIP2, BIP2 and BZIP50 (a transcription factor involved in ER stress response), and BZIP50 and HSFA3 (a transcriptional regulator that specifically binds DNA of heat shock promoter elements). Moreover, the protein information involved in the figures was described as shown in **Supplementary Table S4**. Overall, these results suggest that NAC MTFs, including ZmNAC074 protein, may interact with UPR- and HSR-associated proteins to enhance stress tolerance.

## Discussion

Plants have developed sophisticated signaling pathways to deal with heat stress (Guihur et al., 2022). Numerous NAC TF genes from various plants, such as *ANAC042* and *NAC019* from *Arabidopsis* (Shahnejat-Bushehri et al., 2012; Guan et al., 2014), *OsNTL3* and *SNAC3* from rice (Fang et al., 2015; Liu et al., 2020), *TaNAC2L* from wheat (Guo et al., 2015), *CaNAC035*, *CaNAC46* and *CaNAC2c* from pepper (Zhang et al., 2020; Cai et al., 2021; Ma et al., 2021), have been verified to respond to heat stress, suggesting their potential functions in heat stress tolerance. However, there are few reports on functional identification of heat resistance of NAC TFs in maize. Thus, in this study, *ZmNAC074* was selected from our previous transcriptome data (Qian et al., 2019) and qRT-PCR analysis in maize response to heat stress to perform further functional verification. Further, physiological and molecular responses of transgenic *Arabidopsis* overexpressing *ZmNAC074* under heat stress treatments were revealed in this study.

### *ZmNAC074* is responsive to various abiotic stresses in maize

Studies have shown that under abiotic stresses, membrane-bound transcription factor (MTF) can be transferred into the nucleus and become an active TF (Seo and Kim, 2008), such as TaNTL1 and OsNTL3 (Liu et al., 2020; Sun et al., 2022). A previous study has shown that ZmNAC074 (also known as ZmNTL1) can be localized to plasma membrane and that the GFP-ZmNTL1-△TM (lacking the transmembrane motif) fusion construct product can be co-localized with nuclear marker (Wang et al., 2016). Moreover, ZmNAC074 protein shares high similarity of amino acid sequence with the reported orthologous OsNTL3 protein in rice, suggesting a similar functional role can exist in between ZmNAC074 and OsNTL3. Therefore, we speculate that ZmNAC074, like OsNTL3, can be localized to the plasma membrane under normal conditions and translocate to the nucleus in response to heat stress or other stresses (Liu et al., 2020). For another, *ZmNAC074* may play a crucial role in response to heat, drought, and salt stress, especially heat stress. In addition, under these three kinds of various stresses, the expression of *ZmNAC074* in leaves was changed highly and significantly compared with other tissues, indicating that *ZmNAC074* may play a vital role through the tissue-specific expression in leaves.

### Overexpression of *ZmNAC074* confers thermotolerance in transgenic *Arabidopsis* by regulating the accumulation of multiple stress-related metabolites

Plants have evolved a variety of morphological, physiological and biochemical mechanisms to cope with heat stress, and the corresponding indicators have been used to evaluate heat resistance (Guihur et al., 2022). Phenotypically, the thermotolerance of transgenic *Arabidopsis* was significantly enhanced after heat stress. The enhancement of the adaptability of these transgenic *Arabidopsis* plants to heat stress was inseparable from the synchronous adaptation changes of external morphology and biochemical levels (Lephatsi et al., 2021). For example, levels of lipid oxidation markers such as MDA are typically reduced in plants designed as heat-resistant (Davey et al., 2005; Alché, 2019), while soluble proteins and proline, a penetrant or a free radical scavenger, increase (Alvarez et al., 2022; Ghosh et al., 2022). Therefore, the overexpression of exogenous *ZmNAC074* may enhance tolerance to heat stress through decreasing lipid peroxidation, maintaining the stability of plant membrane structure and inducing proline and soluble protein biosynthesis to enhance osmotic potential in transgenic *Arabidopsis.*

Furthermore, the photosynthetic organelles of plants are highly sensitive to heat stress treatment and plants with higher chlorophyll content can be considered to exhibit greater heat tolerance (Hu et al., 2020; Muhammad et al., 2020). Carotenoids can be used as a stress-protective metabolite and mitigate harmful stress-induced ROS (Kang et al., 2018; Felemban et al., 2019; Guihur et al., 2022). After heat stress treatment, the content of carotenoids in transgenic *Arabidopsis* was higher than that in WT *Arabidopsis*, probably because the overexpression of *ZmNAC074* in *Arabidopsis* may promote the synthesis of carotenoid production related enzymes under heat stress. However, there was no significant difference in chlorophyll content between WT *Arabidopsis* and transgenic *Arabidopsis* plants under heat stress after 24 h, but the chlorophyll content of transgenic *Arabidopsis* was significantly higher than that of WT *Arabidopsis* after 48 h, which may be due to the slight damage caused by heat stress in a short time was not enough for ZmNAC074 to activate numerous related chlorophyll degradation genes. In any case, further study is deserved to excavate the exact mechanisms underlying the positive role of *ZmNAC074* in regulating heat stress response.

### Overexpression of *ZmNAC074* plays a pivotal role in regulating ROS homeostasis

ROS is similar to a double-edged sword and ROS homeostasis is vital for plant growth, development and acclimatization against stresses (Sachdev et al., 2021). Excessive ROS generated by heat stress are toxic molecules capable of causing oxidative damage to proteins, nucleic acids, carbohydrates, cell membranes, lipids, and other sites of cells (Huchzermeyer et al., 2022). However, plants have evolved an antioxidant defense system including diverse antioxidants like SOD and CAT, which can protect them against oxidative damage (Baxter et al., 2014). Consistently, in this study, H_2_O_2_ levels and O_2_^-^ contents were lower while SOD and CAT activities were higher in transgenic *Arabidopsis* than WT *Arabidopsis* under heat stress treatments, indicating that increased SOD and CAT activities resulted from *ZmNAC074* overexpression in transgenic *Arabidopsis* is associated with heat stress tolerance through ROS-mediated stress responsive signaling pathway.

Further, APXs and GPXs can protect cells from stress-induced oxidative damage and play a key role in plant development and growth (Bela et al., 2015; Li et al., 2019; Singh et al., 2022). In this study, the significantly up-regulated expression of *AtAPX1-8* and *AtGPX1-8* in transgenic *Arabidopsis* suggested that *ZmNAC074* may enhance the ability of antioxidants to scavenge excessive ROS by up-regulating these antioxidant enzyme genes, thus enhancing the ability to resist heat stress. Notably, several genes (*AtAPX3*, *AtAPX7*, *AtAPX8*, and *AtGPX1*) in transgenic *Arabidopsis* were expressed at higher levels than in WT *Arabidopsis* under normal conditions. We speculate that *ZmNAC074* may trigger antioxidant defense system through signaling before heat stress. Under heat stress treatments, the antioxidant response was elevated, further increasing the activity of antioxidant enzymes. This protective mechanism may appear to cause more limited damage to cellular components in transgenic *Arabidopsis* lines in comparison to WT *Arabidopsis* plants. In conclusion, these results have evidenced that overexpression of *ZmNAC074* can trigger the expression levels of numerous stress-associated genes in transgenic *Arabidopsis*. These genes can be considered as direct or indirect downstream genes of *ZmNAC074*, and further works are required to reveal the signal transduction pathways associated with *ZmNAC074* during heat stress.

### Overexpression of *ZmNAC074* regulates the expression of HSR-associated genes in transgenic *Arabidopsis*

Previous studies have demonstrated that heat shock response (HSR) in cytoplasm and unfolded protein response (UPR) in ER act as two evolutionarily conserved systems that protect plants from heat stress (Li et al., 2020b). Heat stress induces HSR, in which heat shock transcription factors (HSFs) are a class of conservative transcription factors and can bind to heat shock cis-regulatory elements in promoter regions of *HSP* genes and promote the expression of *HSP* genes (Hayes et al., 2021; Li and Howell, 2021). HSPs, as molecular chaperones, promote the correct folding and aggregation of proteins (Hayes et al., 2021). In this study, the expression levels of most *HSP* and *HSF* genes in transgenic *Arabidopsis* were more highly expressed in the transgenic *Arabidopsis* lines, indicating that overexpression of *ZmNAC074* confers enhanced heat stress tolerance of transgenic *Arabidopsis* by up-regulating the expression levels of these heat stress responsive genes. Therefore, *ZmNAC074* may be a candidate gene for genetic engineering in generating crops to enhance heat tolerance.

### Overexpression of *ZmNAC074* regulates the expression of *AtbZIP60* (UPR-associated gene) in transgenic *Arabidopsis*

Previous studies have revealed that *AtbZIP60* can encode an UPR-associated transcription factor, and respond to NaCl and tunicamycin (TM) and thereby induce endoplasmic reticulum (ER) stress and enhance the abiotic stress tolerance in *Arabidopsis* (Li et al., 2017). Moreover, the close relationship between plant NAC proteins and ER stress has been revealed (Yu et al., 2022). For instance, three *Arabidopsis* NAC TFs (including AtNAC062, AtNAC089, and AtNAC103) can be activated to respond to ER stress and regulate different UPR-associated gene clusters (Sun et al., 2013; Yang et al., 2014a; Yang et al., 2014b). Notably, it has been reported that OsNTL3 (orthologous with ZmNAC074) directly binds to the promoter of *OsbZIP74* (orthologous with *AtbZIP60*) and regulates its expression in response to heat stress (Liu et al., 2020). Thus, in this study, the expression level of *AtbZIP60* in transgenic *Arabidopsis* was significantly higher than that in WT *Arabidopsis* under heat stress, indicating that *ZmNAC074* may act on *AtbZIP60* to alleviate UPR caused by heat stress to enhance heat stress tolerance. Moreover, the protein protein interaction networks further revealed that AtNAC089 (orthologous with ZmNAC074) interacted with AtbZIP60 in *Arabidopsis* and OsNTL3 may also affect OsbZIP50 (orthologous to AtbZIP60) by interacting with BiP2 or BiP4 in rice, implying that ZmNAC074 may function in heat stress-induced UPR-associated regulatory pathways. However, further investigations are needed to clarify the detailed mechanisms of the interactions between ZmNAC074 and these bZIP proteins in regulating stress responses in maize.

**Figure 8:**
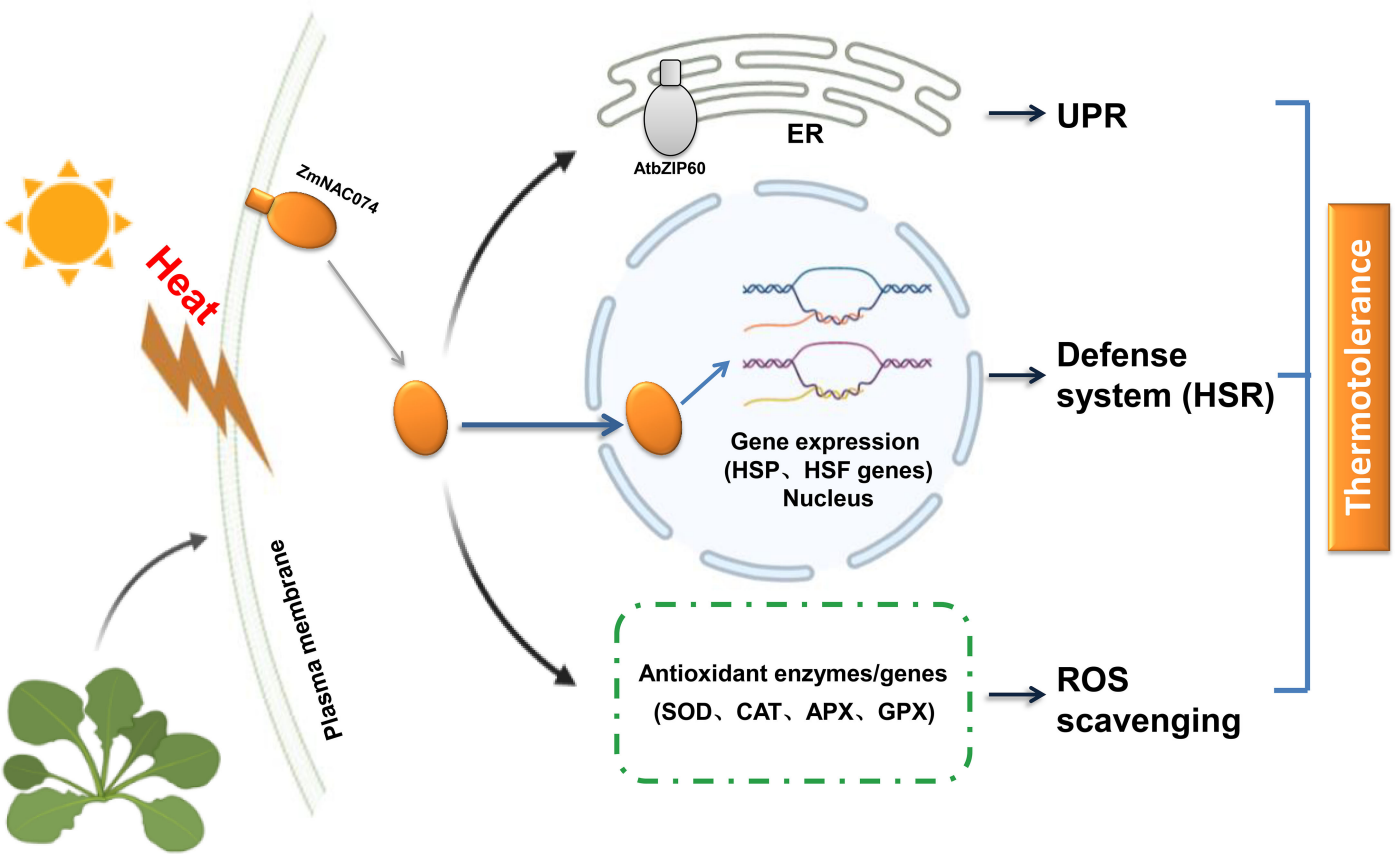
A proposed model for the potential roles of *ZmNAC074* in response to heat stress in transgenic *Arabidopsis.* Under normal conditions, the putative protein, ZmNAC074, is located on the plasma membrane. However, under heat stress treatments, ZmNAC074 protein may be treated by an unknown mechanism to relocate the activated ZmNAC074 protein from the plasma membrane to the nucleus and up-regulate the expression of *AtbZIP60* that associated with UPR and other downstream genes that associated with HSR and ROS. Therefore, ZmNAC074 protein may transfer heat stress signals from the plasma membrane to the nucleus and regulate heat stress-related genes to confer enhanced thermotolerance in transgenic *Arabidopsis.*.

Taken together, we successfully constructed transgenic *Arabidopsis* plants overexpressing *ZmNAC074* under the control of *CaMV 35S* promoter in this study. As expected, the transgenic *Arabidopsis* plants exhibited enhanced tolerance to heat stress, which might be associated with modulating HSR, UPR and ROS homeostasis as shown in the proposed model in this study **(** **Figure 8** **)**. Therefore, overexpression of *ZmNAC074* may confer thermotolerance in transgenic *Arabidopsis* through the involvement in multiple diverse regulatory pathways. However, the molecular mechanisms underlying heat stress response still remain to be further elucidated.

## Conclusion

In conclusion, our results in this study demonstrate that *ZmNAC074* encodes a maize stress- responsive NAC transcription factor, which may be involved in response to various abiotic stresses such as heat, drought and salt. Moreover, functional analysis displays that overexpression of *ZmNAC074* can confer enhanced tolerance to heat stress through modulating the alterations of various stress-related metabolites including reactive oxygen species (ROS), antioxidant, malondialdehyde (MDA), proline, soluble protein, chlorophyll and carotenoid contents in transgenic *Arabidopsis*, indicating that *ZmNAC074* may play a crucial role in enhancing heat tolerance of transgenic *Arabidopsis* through the involvement in several different metabolic pathways. Further, the expression analyses of some ROS scavenging genes, HSR- and UPR-associated genes demonstrate that *ZmNAC074* can enhance heat resistance in transgenic *Arabidopsis* through modulating HSR, UPR and ROS homeostasis. Taken together, our results demonstrate that *ZmNAC074* may encode a functional TF in maize and act as a key candidate regulatory gene for heat stress tolerance regulation and genetic improvement in maize as well as in other crops.

## Data availability statement

The datasets presented in this study can be found in online repositories. The names of the repository/repositories and accession number(s) can be found in the article/**Supplementary Material**.

## Ethics statement

This article does not contain any studies with human participants or animals performed by the authors. These methods were carried out in accordance with relevant guidelines and regulations including the IUCN Policy Statement on Research Involving Species at Risk of Extinction and the Convention on the Trade in Endangered Species of Wild Fauna and Flora. We confirm that all experimental protocols were approved by Anhui Agriculture University and Anhui Normal University.

## Author contributions

These studies were designed by YQ. YX, QL, YZ and XL carried out all the experimental analyses and prepared all figures and tables. The manuscript was drafted by YX. YQ assisted in explaining the results and revised the final manuscript. All authors have reviewed and approved the final manuscript.

## Funding

This study was supported by grants from the National Natural Science Foundation of China (NSFC) (Grant NO.31571673) and the open fundings of National Engineering Laboratory of Crop Stress Resistance Breeding (Grant NO.KNZJ1023) and Anhui Provincial Key Laboratory of the Conservation and Exploitation of Biological Resources (Grant NO.Swzy202003) and Anhui Provincial Academic Funding Project for Top Talents in Disciplines (Majors) (Grant NO.gxbjZD 2021044). The funders had no role in the study design, collection, analysis and interpretation of data, or in the writing of the report or decision to submit the article for publication.

## Acknowledgments

The authors are grateful to the editor and reviewers for critically evaluating the manuscript and providing constructive comments for its improvement.

## Conflict of interest

The authors declare that the research was conducted in the absence of any commercial or financial relationships that could be construed as a potential conflict of interest.

## Publisher’s note

All claims expressed in this article are solely those of the authors and do not necessarily represent those of their affiliated organizations, or those of the publisher, the editors and the reviewers. Any product that may be evaluated in this article, or claim that may be made by its manufacturer, is not guaranteed or endorsed by the publisher.
