# Supplementary material for: ZmNAC074, a maize stress-responsive NAC transcription factor, confers heat stress tolerance in transgenic Arabidopsis
Source: Front Plant Sci. 2022 Sep 29;13:986628. doi: 10.3389/fpls.2022.986628 (PMC9558894; doi:10.3389/fpls.2022.986628)
Supplement: Supplementary file 1 [file DataSheet_1.docx]

Supplementary Material

**1 Supplementary Tables**

**Supplementary Table 1 Primer sequences for RT-qPCR**

| **Name** | **Gene ID** | **Primer** | **Amplicon size (bp)** |
| --- | --- | --- | --- |
| *ZmNAC074* | Zm00001d040318 | Forward: 5'- GTTAGGATTCGTTGCGTTTCTT-3' | 107 |
|  |  | Reverse: 5'- CTTCTTCAGGACGTCATAGGAA-3' |  |
| *ZmActin* | Zm00001d010159 | Forward: 5'- GCATCTACATCTATACGCTCGA-3' | 241 |
|  |  | Reverse: 5'- CTGTAACAGACCAAAGTTCAGC-3' |  |
| *AtHSP70-4* | AT3G12580 | Forward: 5'-TACAACATGAGGAACACGATCA-3' | 100 |
|  |  | Reverse: 5'-ATTCAATAGCTTGGTCGATTGC-3' |  |
| *AtHSP101* | AT1G74310 | Forward: 5'-GTATGGTGCTAGGCCTATAAGG-3' | 115 |
|  |  | Reverse: 5'-GCCTGCATCTATGTAAACAGTG-3' |  |
| *AtHSP18.2* | AT5G59720 | Forward: 5'-GGTTGAGGTTGAAGACAAGAAC-3' | 135 |
|  |  | Reverse: 5'-TTCTCCGGTAACCTAAACCTTC-3' |  |
| *AtHSFa1a* | AT4G17750 | Forward: 5'-CAGCGACTTTACTTAATGCGAA-3' | 124 |
|  |  | Reverse: 5'-ACAATGAAGCTATTGTTCGTCG-3' |  |
| *AtHSFa1b* | AT5G16820 | Forward: 5'-CCCAAGTATTTCAAGCACAACA-3' | 131 |
|  |  | Reverse: 5-'AGTAGTTGTTTTCGGCCTCTAA-3' |  |
| *AtHSFa1d* | AT1G32330 | Forward: 5'-GGTAATGGTACGTCTACTACCG-3' | 142 |
|  |  | Reverse: 5'-CCGAAACATTTTCAGGAGTTGT-3' |  |
| *AtHSFA1E* | AT3G02990 | Forward: 5'-TACTAAAGAGTATTGTCCGGCG -3' | 121 |
|  |  | Reverse: 5'-ACTTCCTCTTCGAGTCCAAATT-3' |  |
| *AtHSFA2* | AT2G26150 | Forward: 5'-ATGGGTTTGCAGAATGTGAATC-3' | 174 |
|  |  | Reverse: 5'-CATAGCTGCAACTTGACTCTTG-3' |  |
| *AtHSfA3* | AT5G03720 | Forward: 5'-GCTCTCTTTCTTGGCTAAGTTG-3' | 127 |
|  |  | Reverse: 5'-GTGGTGCTTGATGAACTTCTTT-3' |  |
| *AtHSfA7a* | AT3G51910, | Forward: 5'-AACACTGACCACATCGTATCTT-3' | 81 |
|  |  | Reverse: 5'-GAGAATCGTGGAGAAAGAATGC-3' |  |
| *AtbZIP60* | AT1G42990 | Forward: 5'- GGCAAATGAAGTTTACTCCCAG-3' | 130 |
|  |  | Reverse: 5'-AGCAGGGAACCCAACAGCAGACT-3' |  |
| *AtAPX1* | AT1G07890 | Forward: 5'-GATGTCTTTGCTAAGCAGATGG-3' | 140 |
|  |  | Reverse: 5'-GAGTTGTCGAAGATTAGAGGGT-3' |  |
| *AtAPX2* | AT1G77490 | Forward: 5'-GAAATAGTTGCCTTGTCTGGTG-3' | 134 |
|  |  | Reverse: 5'-CATTTCACTGTCCATGACTGTC-3' |  |
| *AtAPX3* | AT3G09640 | Forward: 5'--AAGGCTCTTCTTGATGATCCTC-3' | 89 |
|  |  | Reverse: 5'-GCTTCCGTATAGTCTTCGAAGA-3' |  |
| *AtAPX4* | AT4G08390 | Forward: 5'-TGAGACGAAGTACACGAAAGAA-3' | 200 |
|  |  | Reverse: 5'-CCTGATCTGCAGCATACTTTTC-3' |  |
| *AtAPX5* | AT4G09010 | Forward: 5'-TTGCTTTCAACCACCATTAAGG-3' | 240 |
|  |  | Reverse: 5'-TGCATATGAAATAGGACCTCCC-3' |  |
| *AtAPX6* | AT4G32320 | Forward: 5'-GACTGCTTCTCTGGTTAAGACT-3' | 205 |
|  |  | Reverse: 5'-CCTTCTACTTGCAACGAAAACA-3' |  |
| *AtAPX7* | AT4G35000 | Forward: 5'-GCAAAGGATGAAGATGCATTCT-3' | 187 |
|  |  | Reverse: 5'-TCCGGATCTCGTAAAAGTAACC-3' |  |
| *AtAPX8* | AT4G35970 | Forward: 5'-GAACTCTCTTCTCTCGTATGGG-3' | 206 |
|  |  | Reverse: 5'-TAGAGCCTTGTCGGTTTTAAGT-3' |  |
| *AtGPX1* | AT1G63460 | Forward: 5'-TTCCAAATGTGGGATGACAAAC-3' | 177 |
|  |  | Reverse: 5'-GGGAATTCAGATTTGAAGCGAG-3' |  |
| *AtGPX2* | AT2G25080 | Forward: 5'-TCTCTTCAAGTCTCGTCCTTTC-3' | 164 |
|  |  | Reverse: 5'-ATGTCAAACCACATCTTGAAGC-3' |  |
| *AtGPX3* | AT2G31570 | Forward: 5'-GGACCAATACAAAGGCAAAACT-3' | 198 |
|  |  | Reverse: 5'-GTGCAGACAGTTTGTTGAATCT-3' |  |
| *AtGPX4* | AT2G43350 | Forward: 5'-AATGTAGCCTCTAAGTGTGGTC-3' | 270 |
|  |  | Reverse: 5'-CCCAAACAATCCTCCTTTTTGT-3' |  |
| *AtGPX5* | AT2G48150 | Forward: 5'-CAAAACGCAGCACCAATCTATA-3' | 155 |
|  |  | Reverse: 5'-TTCTCGATTGATAGCGGTGTAA-3' |  |
| *AtGPX6* | AT3G63080 | Forward: 5'-AAGAGGCTCATCAATTTGCTTG-3' | 204 |
|  |  | Reverse: 5'-TTGTGCCATAACGATCAATGAC-3' |  |
| *AtGPX7* | AT4G11600 | Forward: 5'-GGAATCAAGAGCCTGGTACTAA-3' | 104 |
|  |  | Reverse: 5'-TTTGTCACCGTTAACATCAACC-3' |  |
| *AtGPX8* | AT4G31870 | Forward: 5'-GCAGAGTTCCCTATATTCGACA-3' | 156 |
|  |  | Reverse: 5'-CTCGACAACTTTGCCCTTTTTA-3' |  |
| *AtActin* | AT3G18780 | Forward: 5'- CTCCTTTGTTGCTGTTGACTAC-3' | 248 |
|  |  | Reverse: 5'- GCACAATGTTACCGTACAGATC-3' |  |

**Supplementary Table 2 Information of *NAC* genes in plants**

| **Name** | **Gene ID** |
| --- | --- |
| AtNAC89 | AT5G22290.1 |
| AtNAC60 | AT3G44290.1 |
| AtNTL8 | AT2G27300.1 |
| ZmNAC28 | Zm00001d027395 |
| OsNTL3 | LOC_Os01g15640.1 |
| AtNAC103 | AT5G64060 |
| ZmNAC074 | Zm00001d040318 |
| AtNAC62 | AT3G49530.1 |
| AtNAC91 | AT5G24590.2 |
| AtNAC14 | AT1G33060.2 |
| ZmNAC130 | Zm00001d045617 |
| OsNAC19 | LOC_Os06g01230 |
| ZmNAC145 | Zm00001d024543 |

**Supplementary Table 3 The amino acid sequences of conserved motifs**

| **Motif** | **Motif Consensus** |
| --- | --- |
| 1 | DSEWFFFCPRDRKYPNGSRSNRATVAGYWKATGKDRKIKSG |
| 2 | IGTKKTLVFHRGRAPRGERTEWIMHEYRITEKELDATKVGQ |
| 3 | ALPPGFRFHPTDEELISYYLKRKINGREN |
| 4 | IPEVDIYKFEPWDLPDKSVIK |
| 5 | YVLCRLFKKN |
| 6 | IKKGCGKFMRSKNRTGFIFKKIAAMGCSYGGLFRVGVVAVVCLMSVCSL |
| 7 | QLPDLEPEQRFDGFPNITSPIRPYSDHPFFGNAGEQDLSAHFGSIJSEQD |
| 8 | QGTAQRRLRLQTNLN |
| 9 | PLPDMIDKQMQSLLZERPLQKDKGEENNESLSNCFIGJYSIKSINKARWD |
| 10 | QVDSDEDFYADILRDEIIKLD |

**Supplementary Table 4 Information of proteins in Protein Protein Interaction networks**

| **Protein** | **Information** |
| --- | --- |
| BZIP28 | Basic-leucine zipper (bZIP) transcription factor family protein; Up-regulated in response to heat stress. |
| MC5 | Metacaspase 2b; Metacaspase-5; Cysteine protease that cleaves specifically after arginine or lysine residues. |
| CPK32 | Calcium-dependent protein kinase 32; Belongs to the protein kinase superfamily. Ser/Thr protein kinase family. CDPK subfamily. |
| AT2G47780 | Rubber elongation factor protein (REF); Belongs to the REF/SRPP family. |
| BZIP60 | Basic region/leucine zipper motif 60; AtbZIP60 consists of a bZIP DNA binding domain followed by a putative transmembrane domain. |
| CZF1 | Zinc finger CCCH domain-containing protein 29; Involved in salt stress response. May positively modulate plant tolerance to salt stress. |
| AT5G42050 | DCD (Development and Cell Death) domain protein. |
| RBOHA | Respiratory burst oxidase homolog protein A; Calcium-dependent NADPH oxidase that generates superoxide; Belongs to the RBOH (TC 5.B.1.3) family. |
| IRE1A | Serine/threonine-protein kinase/endoribonuclease IRE1a; Senses unfolded proteins in the lumen of the endoplasmic reticulum via its N-terminal domain which leads to enzyme auto- activation. |
| TIP | Arabidopsis nac domain containing protein 91; Transcription activator essential for the anti-viral defense called virus basal resistance response pathway Not involved in HRT-mediated hypersensitive response (HR) and resistance to TCV . |
| TET8 | Tetraspanin-8; May be involved in the regulation of cell differentiation. |
| AT5G43460 | HR-like lesion-inducing protein-related. |
| TSPO | TSPO(outer membrane tryptophan-rich sensory protein)-related; Encodes a membrane-bound protein designated AtTSPO (Arabidopsis thaliana TSPO-related). |
| NAC062 | NTM1 (NAC WITH TRANSMEMBRANE MOTIF 1)-LIKE 6; Transcriptional activator activated by proteolytic cleavage through regulated intramembrane proteolysis (RIP) Transcriptional activator involved in response to cold stress. |
| IRE1-1 | Serine/threonine-protein kinase/endoribonuclease IRE1b; Senses unfolded proteins in the lumen of the endoplasmic reticulum via its N-terminal domain which leads to enzyme auto- activation. |
| NAC089 | Fructose-sensing quantitative trait locus 6; NAC domain containing protein 89; Transcription factor involved in plant cell division. |
| CPK28 | Calcium-dependent protein kinase 28; May play a role in signal transduction pathways that involve calcium as a second messenger (Probable). Acts as developmentally controlled regulator for coordinated stem elongation and vascular development. |
| WRKY33 | Probable WRKY transcription factor 33; Transcription factor. Interacts specifically with the W box (5'-TTGAC[CT]-3'), a frequently occurring elicitor-responsive cis-acting element. |
| VAP27-1 | Vamp/synaptobrevin-associated protein 27-1; Encodes VAP27 (for Vesicle-Associated Protein). |
| WRKY40 | Probable WRKY transcription factor 40; Pathogen-induced transcription factor. Binds W-box sequences in vitro. Forms protein complexes with itself and with WRKY40 and WRKY60. |
| BAG7 | BAG family molecular chaperone regulator 7; A member of Arabidopsis BAG (Bcl-2-associated athanogene) proteins, plant homologs of mammalian regulators of apoptosis. |
| AT4G29780 | Uncharacterized protein At4g29780. |
| NAC103 | NAC domain containing protein 103 (NAC103); Its function is described as sequence-specific DNA binding transcription factor activity. |
| AT5G15660 | F-box and associated interaction domains-containing protein. |
| AT1G27300 | Uncharacterized protein At1g27300. |
| AT4G22960 | Ubiquitin carboxyl-terminal hydrolase mindy-1/2. |
| AT2G32380 | Transmembrane protein 97, predicted. |
| SZF1 | Zinc finger CCCH domain-containing protein 47; Involved in salt stress response. May positively modulate plant tolerance to salt stress. |
| TI1 | Trypsin inhibitor protein 1; Member of the defensin-like (DEFL) family. |
| AT1G04340 | HR-like lesion-inducing protein-related;. |
| BZIP17 | Basic-leucine zipper (bzip) transcription factor family protein; Transcriptional activator involved in salt and osmotic stress responses. Functions as a stress sensor and transducer in a signaling pathway that resembles an ER stress response. |
| AT1G47370 | Toll-Interleukin-Resistance (TIR) domain family protein; Its function is described as transmembrane receptor activity; Involved in signal transduction, defense response, innate immune response; Located in intrinsic to membrane. |
| IRE1 | Serine/threonine-protein kinase/endoribonuclease IRE1; Involved in endoplasmic reticulum (ER) stress response. |
| BIP2 | Heat shock 70 kDa protein BIP2; Functions as chaperone during endoplasmic reticulum (ER) stress response. |
| Q9AWZ5 | Os01g0225600 protein. |
| HSFA1 | Heat stress transcription factor A-1; Transcriptional regulator that specifically binds DNA of heat shock promoter elements (HSE); Belongs to the HSF family. |
| PIP2-1 | Probable aquaporin PIP2-1; Aquaporins facilitate the transport of water and small neutral solutes across cell membranes. |
| BIP1 | Heat shock 70 kDa protein BIP1; Functions as a sensor of the ER stress response, and provides suitable conditions for the production of secretory proteins by alleviating ER stress; Belongs to the heat shock protein 70 family. |
| HSFA9 | Heat stress transcription factor A-9; Transcriptional regulator that specifically binds DNA of heat shock promoter elements (HSE); Belongs to the HSF family. |
| TIP1-2 | Probable aquaporin TIP1-2; May be involved in transport from the vacuolar compartment to the cytoplasm (By similarity). |
| Q6Z0A5 | cDNA clone:J023093J22, full insert sequence. |
| Q2R2L6 | Myb-like DNA-binding domain containing protein. |
| BZIP39 | bZIP transcription factor 39; Transcription factor involved in endoplasmic reticulum (ER) stress response. Acts as ER stress sensor and activates the transcription factor BZIP50 and the chaperone BIP1. |
| OsNTL3 | NAC domain-containing protein 74; Probable transcription factor involved in stress response. |
| ERDJ3B | DnaJ protein ERDJ3B; May play a role in protein folding in the endoplasmic reticulum. |
| BZIP60 | bZIP transcription factor 60; Transcription factor involved in endoplasmic reticulum (ER) stress response. Acts as ER stress sensor and activates the transcription factor BZIP50 and the chaperone BIP1. |
| GYRA | Probable DNA gyrase subunit A, chloroplastic/mitochondrial; Belongs to the type II topoisomerase GyrA/ParC subunit family. |
| BZIP50 | bZIP transcription factor 50; Transcription factor involved in endoplasmic reticulum (ER) stress response . Acts downstream of the ER stress sensors IRE1, BZIP39 and BZIP60 to activate BiP chaperone genes. |
| PIP1-2 | Probable aquaporin PIP1-2; Aquaporins facilitate the transport of water and small neutral solutes across cell membranes. |
| BIP4 | Heat shock 70 kDa protein BIP4; Functions as chaperone during endoplasmic reticulum (ER) stress response. |
| HSFA3 | Heat stress transcription factor A-3; Transcriptional regulator that specifically binds DNA of heat shock promoter elements (HSE). |
